# Spermidine and Eugenol Modulate Tight Junction and Stemness Markers in Colorectal Cancer Spheroids

**DOI:** 10.3390/ijms27062894

**Published:** 2026-03-23

**Authors:** Silvia Dilloo, Silvana Hrelia, Cristina Angeloni, Marco Malaguti, Giovanni Dinelli, Francesca Truzzi

**Affiliations:** 1 Department of Agricultural and Food Sciences, Alma Mater Studiorum—University of Bologna, 40127 Bologna, Italy; silvia.dilloo2@unibo.it (S.D.); giovanni.dinelli@unibo.it (G.D.); 2 Department for Life Quality Studies, Alma Mater Studiorum—University of Bologna, 47921 Rimini, Italy; silvana.hrelia@unibo.it (S.H.); cristina.angeloni@unibo.it (C.A.); marco.malaguti@unibo.it (M.M.)

**Keywords:** colorectal cancer, cancer spheroids, spermidine, eugenol, cancer stem cells, tight junctions, Occludin, Zonula occludens-1 (ZO-1), ALDH1A1, SOX2, VE-cadherin, CD133

## Abstract

Alterations in tight junction (TJ) organization and dysregulation of cancer stem cell (CSC)-associated markers are increasingly recognized as molecular features linked to colorectal cancer (CRC) progression, heterogeneity and clinical outcome. Bioactive dietary compounds such as spermidine (SPD) and eugenol (EUG) have been proposed as modulators of cancer-related molecular pathways; however, their combined effects on CRC spheroid models relevant to molecular characterization remain insufficiently defined. In the present study, the molecular impact of SPD and EUG, administered individually or in combination, was evaluated in primary and metastatic CRC spheroids. First-generation spheroids derived from Caco-2 and SW620 cells were exposed to SPD, EUG, or SPD+EUG at the time of seeding, and spheroid growth and self-renewal capacity were monitored across successive generations. The expression of TJ- and CSC-associated markers was assessed at both the transcript and protein levels using reverse transcription–quantitative polymerase chain reaction (RT-qPCR), Western blotting and immunohistochemistry. The combined SPD+EUG treatment was associated with a marked reduction in spheroid area and self-renewal capacity in both CRC models. Baseline molecular profiling revealed higher TJ marker expression in Caco-2 spheroids and enrichment of CSC-associated markers in SW620 spheroids. Treatment-induced modulation of CSC- and TJ-related transcripts was observed; however, transcript-level changes were not consistently mirrored at the protein level, indicating the involvement of post-transcriptional regulatory mechanisms. In particular, Occludin (OCLN), Zonula occludens-1 (ZO-1), CD133, ALDH1A1, SOX2 and VE-cadherin exhibited divergent RNA and protein expression patterns depending on cell type and treatment condition. Collectively, these findings underscore the relevance of three-dimensional CRC spheroid models for molecular profiling studies and highlight the importance of integrating transcript- and protein-level analyses when evaluating bioactive compounds with potential diagnostic and translational relevance in colorectal cancer.

## 1. Introduction

Based on updated estimates from the International Agency for Research on Cancer, colorectal cancer (CRC) is the third-most common cancer and the second leading cause of cancer-related deaths [1]. In CRC, of which adenocarcinomas account for approximately 96%, surgery, chemotherapy, and radiotherapy represent the main clinical therapies and have proven effective in eliminating differentiated CRC cells that form the bulk of the tumor mass [2,3]. CRC is a multifactorial disease influenced by genetic, environmental, and lifestyle factors. Most cases are sporadic and develop gradually through a multistep adenoma–carcinoma sequence driven by accumulated genetic mutations, leading to altered signaling pathways, increased tumor cell proliferation, and resistance to apoptosis [4].

For a substantial portion of CRC patients, traditional therapies are insufficient to eradicate a minority subpopulation of highly tumorigenic cancer stem cells (CSCs), responsible for the initiation, growth, progression, therapy resistance and recurrence of CRC [2,5,6,7,8].

In addition, colorectal CSCs (CCSCs) are present both in primary non-invasive (containment in the colon) and invasive type tumors (spread to surrounding tissues), as well as in secondary or metastatic tumors (spread to distant parts of the body).

CSCs, comprising 0.3 to 2.2% of the CRC mass [9], are distinguishable based on the presence of unique functional cell surface/intracellular markers and the “stemness characteristics” of self-renewal and pluripotency [7,10]. CCSCs can arise from somatic cells and stem/progenitor cells, as well as from the dedifferentiation (reprogramming) of non-CCSCs cells in the tumor microenvironment (TME) [7,9,10,11,12]. The complex interplay between genetic, epigenetic and TME alterations is implicated in the formation of CCSCs from the respective cell sources. In turn, CCSCs and the TME engage in a dynamic interaction that influences signaling pathways and regulates the expression of tight junction (TJ) proteins, ultimately impacting CCSC survival, self-renewal and resistance [13].

Within the framework of the classic CRC development pathway, the initiation, promotion, progression, and metastasis steps are, respectively, associated with mutations in the *APC* suppressor gene, *KRAS* proto-oncogene, *DCC* suppressor gene and the *TP53* suppressor gene [10]. Each of the aforementioned mutations negatively impacts various signal transduction pathways, promoting CCSC formation. Of particular interest is the *APC* mutation-based activation of the Wingless integrated/Beta-catenin (Wnt/β-catenin) pathway, implicated as the central mechanism driving colorectal carcinogenesis in 80% of CRCs [9,10,14]. Dysfunctional Wnt/β-catenin signaling causes increased β-catenin accumulation and, together with aberrant cross-communication with upregulated Notch and Hedgehog pathways (and additional signaling pathways), collectively drive CCSC cell surface marker-associated (CD133, CD144, CD24, CD166, CD44, CD29, LGR5) self-renewal and tumorigenesis properties. Additionally, these signaling pathways promote the expression of pluripotent intracellular transcription markers (OCT4, SOX2) and non-CCSC cell dedifferentiation into CCSCs during adenoma/carcinoma development [2,6,10,14].

Given that the plasticity of CCSCs and the functionality of their markers underlie carcinogenesis and therapeutic resistance, targeting and eradicating these cells is unanimously considered an important objective for future research [2,7,9,10,11,12,14]. Due to the emerging roles of TJ proteins in cancer, there is also growing interest in targeting TJ expression in primary and metastatic tumors [15,16]. In particular, in metastatic tumors, the loss of TJs is a feature of epithelial–mesenchymal transition (EMT), a process promoting invasiveness and metastasis, which is also linked to the acquisition of CSC characteristics [16,17,18,19].

Therapeutic strategies aimed at eliminating CCSCs in the clinical stages of CRC are primarily aimed at targeting cell surface markers and signal transduction pathways (specifically the Wnt/β-catenin pathway), and include the use of small molecule inhibitors, monoclonal antibodies, immunotherapy, natural products and combination therapies [6,9,10,14]. Instead, in the preclinical stages of CRC (initiation and promotion) and in combatting tumor recurrence, there has been increasing interest in the efficacy of natural products in targeting cell surface markers and signal transduction pathways [6,19,20,21]. Natural products are of increasing interest in targeting CRCs, attributable to the presence of biologically active components able to simultaneously influence multiple signaling pathways with negligible side effects.

In our previous work [22], the efficacy of exogenous supplementation with wheat germ spermidine (SPD), alone and in combination with clove eugenol (EUG), was shown for the first time to reduce spheroid growth, including vitality, surface area, necrotic area and apoptosis, in homotypic spheroids using both a lymph node metastatic CRC line (SW620) and an extensively used, non-invasive primary tumor cell line (Caco-2). Interestingly, the combination of SPD and EUG (SPD+EUG) produced a synergistic effect in reducing the migration percentage of SW620 cells [22].

EUG, chemically identified as 4-allyl-2-methoxyphenol or 2-methoxy-4-(2-propenyl)phenol, is a phenolic aromatic compound primarily derived from clove oil (*Syzygium aromaticum* L., syn. *Eugenia caryophyllata*, Family Myrtaceae) that exhibits significant antioxidant, anti-inflammatory, analgesic, antimicrobial, neuroprotective, and hepatoprotective properties beyond its anti-cancer potential [23,24,25]. Similarly, SPD is a ubiquitous natural polyamine characterized by geroprotective features that promote longevity, enhance cognitive function and memory, and provide notable anti-inflammatory, antioxidant, and cardioprotective activities [26,27,28]. While EUG is well-recognized for its potent anti-cancer potential, research on SPD shows conflicting results, with most studies reporting tumor-promoting effects and emerging studies indicating anti-tumorigenic effects. This discrepancy led to the development of a recent hypothesis by Zimmermann et al. [27], stating that exogenous supplementation of SPD elicits anti-tumorigenic responses that override the stimulation of cancer proliferation promoted by endogenously produced polyamines (specifically SPD). The underlying anti-CRC effects on tumor growth and metastasis by exogenous SPD administration, both alone and in combination with EUG, as demonstrated by Dilloo et al. [22], warrant further investigation, particularly regarding the preventative anti-CRC potential of exogenous SPD, both alone and in combination with EUG, on CCSC expression and associated TJ protein expression.

The present investigation aimed to analyze the capacity for the formation of secondary and tertiary spheroid generations following a seven-day exposure to SPD, EUG and SPD+EUG in primary-generation spheroids of both SW620 and Caco-2. CSC markers CD133, ALDH1A1, SOX2, and VE-cadherin, as well as TJ proteins occludin (OCLN) and zonula occludens-1 (ZO-1), were measured after seven days of exposure in primary-generation spheroids, with the objective of evaluating the efficacy of and potential interplay between SPD and EUG on CSC markers and TJ protein expression in relation to spheroid formation. EUG has previously been shown to reduce the population of CCSCs (together with invasion and migration) in CRC cells, including SW480 and SW620 [29,30]. To the best of our knowledge, no reports have specifically documented the direct effects of exogenous SPD addition on CSC expression in CRC spheroids in a preventative capacity. Furthermore, there are no reports describing the direct effects of either SPD or EUG on TJ protein expression in CRC spheroids.

## 2. Results

In the present study, homotypic spheroids derived from SW620 and Caco-2 cells were the sole model systems used. Previously, evidence demonstrated that SPD and EUG, administered either individually or in combination, significantly reduced spheroid area by promoting apoptosis [22]. Given that homotypic spheroid cultures are enriched in CSCs and represent robust, reproducible, simple, and cost-effective model systems for preliminary in vitro research [31], investigation of the potential efficacy of SPD (150 µM), EUG (100 µM) and SPD+EUG (150 µM + 100 µM) was extended to include aspects of spheroid self-renewal and associated TJ and CSC gene expression. Comparisons were made with untreated controls (CTRLs).

### 2.1. Formation of Secondary and Tertiary Spheroid Generations Following Spermidine (SPD) and Eugenol (EUG) Treatments of Primary-Generation SW620 and Caco-2 Spheroids

Building on our previous findings demonstrating a synergistic anti-tumorigenic effect of the SPD and EUG combination [22], the first objective was to evaluate if the anti-CRC effects were more effective on established spheroids (to simulate a therapeutic treatment) or whether efficacy was increased when applied prior to spheroid development (reflecting a preventative scenario). For this preliminary pilot phase, only the SPD+EUG combination was utilized to identify the optimal therapeutic window. Hence, SPD+EUG was administered under two experimental conditions: to already formed SW620 and Caco-2 spheroids, 24 h after cell seeding, and concurrently with cell seeding, prior to spheroid development. On day 7 (168 h), the primary spheroids, both untreated (CTRL) and treated with SPD+EUG, were evaluated for spheroid area and viability using the 3-(4,5-dimetiltiazol-2-il)-2,5-difeniltetrazolium (MTT) assay. Thereafter, both the CTRL and SPD+EUG-treated spheroids were reseeded to form second-generation spheroids without further SPD+EUG treatment. Second-generation spheroid area and viability were again evaluated after seven days (168 h).

**Figure 1:**
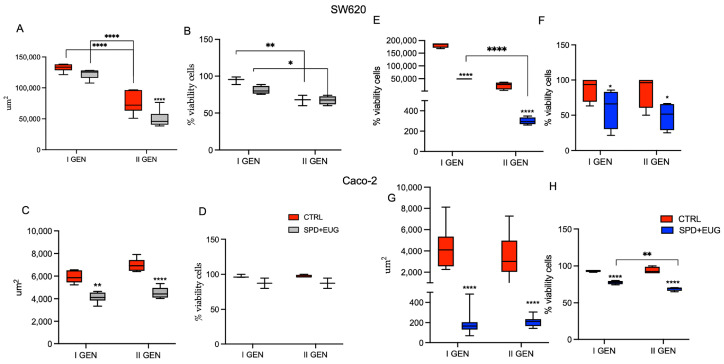
Impact of treatment timing on metastatic SW620 and primary Caco-2 colorectal cancer (CRC) spheroid area and viability. Panels (**A**–**D**) show results for SW620 (**A**,**B**) and Caco-2 (**C**,**D**) when spermidine (SPD) and eugenol (EUG) (SPD+EUG) was administered 24 h post-seeding. Panels (**E**–**H**) show results for SW620 (**E**,**F**) and Caco-2 (**G**,**H**) when treatment was applied at the time of seeding (time zero). Results were evaluated after 168 h for both the primary-generation (I GEN) and the treatment-free secondary generation (II GEN). Data are expressed relative to the untreated control (CTRL). Significant differences: * *p* < 0.05, ** *p* < 0.01, **** *p* < 0.0001.

When SPD+EUG was administered 24 h after cell seeding, no significant differences in spheroid area (Figure 1A) or viability (Figure 1B) were evident between the untreated CTRL and SPD+EUG-treated first-generation SW620 spheroids. The spheroid area of the second-generation CTRLs was significantly smaller compared to the first generation, and the treated spheroids were significantly smaller than the CTRLs (Figure 1A). Viability was not significantly different between the CTRL and SPD+EUG-treated spheroids, but both the CTRL and treated spheroids were significantly less viable in the second generation (Figure 1B). For Caco-2, compared to the respective CTRLs, spheroid area was significantly decreased in the SPD+EUG-treated first-generation and second-generation spheroids (Figure 1C). Viability was not significantly different between the CTRLs and SPD+EUG-treated spheroids in either the first- or second-generations (Figure 1D).

In contrast, when SPD+EUG was administered at the same time of cell seeding (time zero), SW620 spheroid area (Figure 1E) and viability (Figure 1F) were significantly decreased in both first-generation and second-generation spheroids compared to the respective untreated CTRLs. Specifically, the Caco-2 spheroid area was 25% and 1% of the CTRLs in the first- and second-generation-treated spheroids, respectively (Figure 1H). Caco-2 spheroids exposed to SPD+EUG showed significantly reduced areas that were approximately 5% of the respective CTRLs in both the first and second spheroid generations (Figure 1G).

**Figure 2:**
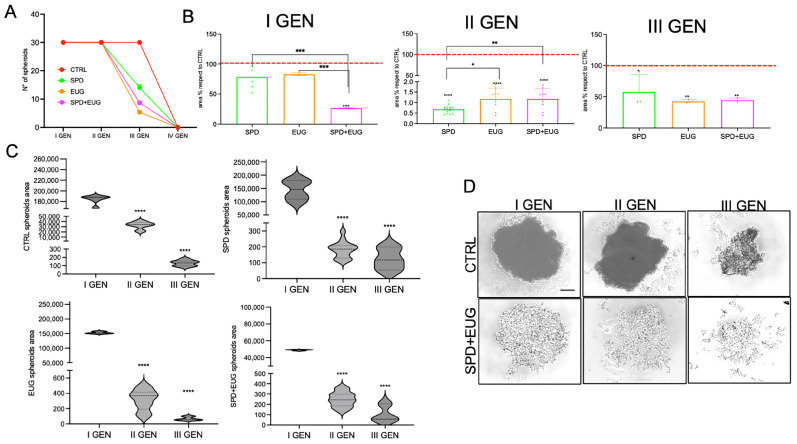
Growth trend and morphology of metastatic SW620 CRC spheroids across consecutive generations (first to fourth, I–IV GEN). (**A**) Total number of spheroids, (**B**) area relative to CTRL, and (**C**) absolute area following a 168 h exposure to SPD, EUG, and SPD+EUG administered at the time of seeding for the first generation (I GEN). The red dashed line in panel B represents the untreated CTRL baseline (100%). All treatments were compared to the untreated CTRL. (**D**) Representative micrographs showing the morphology of CTRL and SPD+EUG-treated spheroids across generations (scale bar = 50 µm). Significant differences: * *p* < 0.05, ** *p* < 0.01, *** *p* < 0.001, **** *p* < 0.0001.

**Figure 3:**
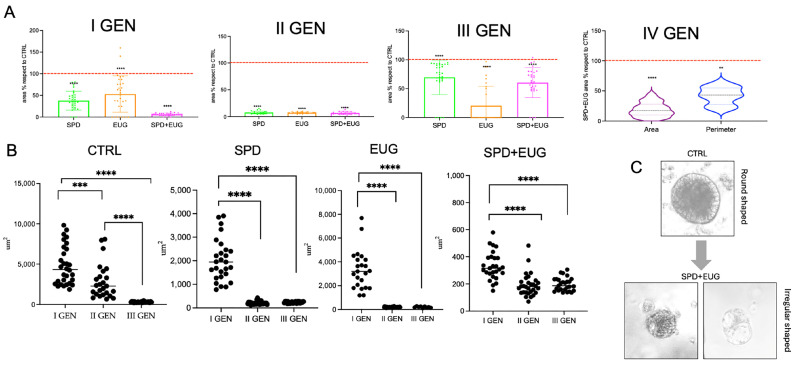
Growth trend and morphology of primary Caco-2 CRC spheroids across consecutive generations (I–IV GEN). (**A**) Area relative to CTRL and (**B**) absolute area distribution following a 168 h exposure to SPD, EUG, and SPD+EUG administered at the time of seeding for the first generation (I GEN). The red dashed line in panel A represents the untreated CTRL baseline (100%). All treatments were compared to the untreated CTRL. (**C**) Representative micrographs showing the morphology of CTRL and SPD+EUG-treated primary spheroids (scale bar = 50 µm). Significant differences: ** *p* < 0.01, *** *p* < 0.001, **** *p* < 0.0001.

Overall, results (Figure 1) evidenced that when SPD+EUG was administered at time zero, there was a greater impact on spheroid formation. In SW620 spheroids, both area and viability were significantly reduced, whereas in Caco-2 spheroids, a reduction in area was observed with no significant change in viability. Based on these preliminary outcomes, it was decided to investigate the preventative approach in greater detail. Therefore, all subsequent experiments were performed using both the individual compounds (150 µM SPD and 100 µM EUG alone) and their combination. Given that the anti-CRC efficacy of a potential preventative treatment can be assessed on the basis of reducing both the number and size of cancer spheroids across consecutive generations, this aspect was first examined in SW620 and then in Caco-2 spheroids, as reported in Figure 2 and Figure 3, respectively.

CTRL SW620 spheroids maintained a 100% self-renewal capacity over three consecutive generations (Figure 2A). For spheroids treated with SPD, EUG and SPD+EUG in the first generation only, spheroid self-renewal capacity was 100% in the second generation but declined in the third generation to 50, 30, and 17%, respectively (Figure 2A). The areas of the first-generation spheroids treated with SPD and EUG alone were not significantly different from the CTRL, whereas the combination treatment resulted in significantly reduced areas (Figure 2B,C). In the second generation, all spheroids that had been exposed to treatments in the first generation were significantly reduced to approximately 4–10% of the CTRL (Figure 2B,C). The CTRL and treated spheroids all showed a significant reduction in area from the first- to second-generation, with the CTRL showing a further significant reduction in area in the third generation (Figure 2C). The areas of the third-generation treated spheroids increased 10-fold from the second generation and were approximately 40–50% of the CTRL (Figure 2B,C). Analyzing the morphology of spheroids (Figure 2D), the SW620 CTRL spheroids appeared rounded and more compact, whereas SPD+EUG-treated spheroids exhibited an irregular and less compact shape. Figure 2D illustrates the significantly smaller area of the spheroids treated with SPD+EUG compared to the CTRL.

Similar to SW620 spheroids, in Caco-2 spheroids, SPD+EUG was the most effective treatment after the first generation, producing a significantly greater reduction in spheroid area compared to the CTRL than SPD or EUG alone (Figure 3A,B). As observed in SW620, CTRL spheroid area in Caco-2 also significantly decreased across consecutive generations (Figure 3B). All second-generation Caco-2 spheroids exposed to treatments were significantly reduced compared to the untreated CTRL, as well as to the respective first-generation treated spheroids (Figure 3A,B). Second-generation and third-generation spheroid areas were equivalent for the respective treatments (Figure 3B), but when areas were expressed in comparison to the CTRL, an increase was observed (Figure 3A). Fourth-generation spheroids exposed to SPD+EUG showed a similar area compared to the CTRL as shown for the third generation (Figure 3A). The CTRL primary-generation Caco-2 spheroid showed a round shape morphology compared to the elongated shape following exposure to SPD+EUG (Figure 3C).

Given the efficacy of the treatments in reducing the size of primary and secondary spheroids, as well as the number of tertiary SW620 spheroids, the question raised was whether and to what extent TJ and CSC expression was altered in primary spheroids following treatment.

### 2.2. Occludin (OCLN) and Zonula Occludens-1 (ZO-1) Expression in Primary-Generation SW620 and Caco-2 Spheroids Following a Seven-Day Exposure to SPD, EUG and SPD+EUG

Our previous research showed that the total OCLN levels in reconstructed 3D intestinal equivalents (comprising a healthy NCM640 enterocyte layer containing embedded Caco-2 cells) increased significantly following exposure to SPD, EUG and SPD+EUG [22]. However, OCLN expression in the spheroids alone was not determined. Given that TJ proteins are crucial players in the regulation of cell proliferation, migration, and differentiation [18], the aberrant expression or loss of TJ proteins in cancer cells has become a focal point for research into potential therapeutic targets and diagnostic markers [15,16]. For this reason, OCLN and ZO-1, distinctive key constituents of TJs, were analyzed using both reverse transcription–quantitative polymerase chain reaction (RT-qPCR) and Western blot analysis. mRNA levels were quantified for the corresponding genes (*OCLN* and *TJP1* for ZO-1). 

**Figure 4:**
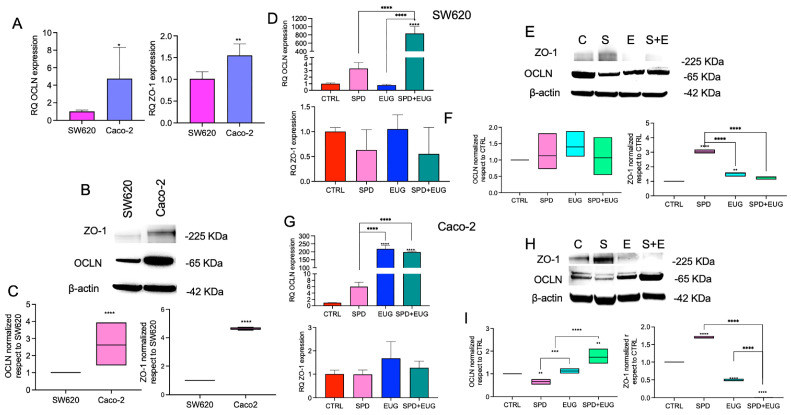
mRNA and protein expression of occluding (OCLN) and zonula occludens-1 (ZO-1) in CRC spheroids. Panels (**A**–**C**) show the baseline comparison of transcript (reverse transcription–quantitative polymerase chain reaction, RT-qPCR) and protein (Western blot) levels between untreated metastatic SW620 and primary Caco-2 spheroids after 168 h (SW620 set as calibrator = 1). Panels (**D**–**I**) illustrate the impact of 168 h exposure toSPD, EUG, and SPD+EUG on mRNA and protein expression in SW620 (**D**–**F**) and Caco-2 (**G**–**I**) models. All results are relative to the untreated CTRL (calibrator = 1). Significant differences: * *p* < 0.05, ** *p* < 0.01, *** *p* < 0.001, **** *p* < 0.0001.

Firstly, comparisons in gene expression were made between untreated CTRL Caco-2 and SW620 spheroids. Relative Quantification (RQ) analysis, using SW620 spheroids as the calibrator (RQ = 1), showed that OCLN and ZO-1 (*TJP1*) mRNA levels were significantly higher in Caco-2 spheroids (Figure 4A). Consistently, protein levels of both OCLN and ZO-1 were also significantly increased in Caco-2 spheroids (Figure 4B,C).

The effect of the treatments was investigated relative to the calibrator CTRL sample. For SW620 spheroids, there was a significant increase in OCLN mRNA levels for the SPD+EUG treatment compared to the CTRL (Figure 4D), whereas no significant differences were observed for ZO-1 (Figure 4F). For Caco-2, both EUG and SPD+EUG induced a significant increase in OCLN mRNA levels compared to the CTRL (Figure 4G). As with SW620, the treatments did not modify ZO-1 RNA transcript expression in Caco-2 spheroids (Figure 4G). Thereafter, protein expression patterns in response to SPD (S), EUG (E) and SPD+EUG (S+E) were evaluated by Western blot analysis and quantified in relation to untreated CTRL (C) spheroids (Figure 4E,F,H,I). For SW620 spheroids (Figure 4E), densitometric quantification (Figure 4F) showed that OCLN protein levels were not significantly modulated by any of the treatments compared to the CTRL. Conversely, ZO-1 protein levels in SW620 cells were significantly higher than the CTRL following exposure to SPD, while no significant differences were observed for the EUG and SPD+EUG treatments (Figure 4F). For Caco-2 spheroids (Figure 4H), protein quantification (Figure 4I) revealed a significant increase in OCLN levels in response to both EUG and the SPD+EUG combination compared to the CTRL. Regarding ZO-1 protein levels in Caco-2 cells, a significant increase was observed following SPD treatment, whereas both EUG and the SPD+EUG combination resulted in significantly lower protein levels than the CTRL (Figure 4I). All target protein bands were normalized to β-actin as the internal reference control.

### 2.3. Cancer Stem Cell (CSC)Gene Expression in Primary-Generation SW620 and Caco-2 Spheroids Following a Seven-Day Exposure to SPD, EUG and SPD+EUG

**Figure 5:**
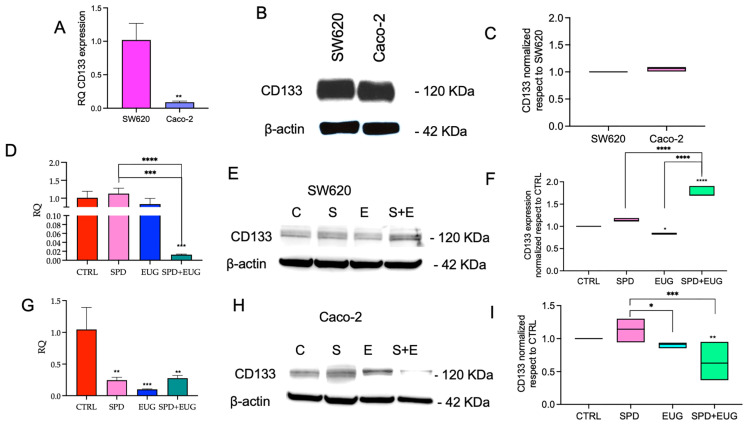
CD133 mRNA and protein expression in CRC spheroids. Panels (**A**–**C**) show the baseline comparison of transcript (RT-qPCR) and protein (Western blot) levels between untreated metastatic SW620 and primary Caco-2 first-generation spheroids after 168 h (SW620 set as calibrator = 1). Panels (**D**–**I**) illustrate the impact of 168 h exposure to SPD, EUG, and SPD+EUG on CD133 expression in metastatic SW620 (**D**–**F**) and primary Caco-2 (**G**–**I**) models. All results are relative to the untreated CTRL (calibrator = 1). Significant differences: * *p* < 0.05, ** *p* < 0.01, *** *p* < 0.001, **** *p* < 0.0001.

The CSC markers analyzed included CD133, ALDH1A1, SOX2 and VE-cadherin. mRNA levels were quantified for the corresponding genes (*PROM1* for CD133, *CDH5* for VE-cadherin, *SOX2*, *ALDH1A1*). CD133, also known as Prominin-1, a transmembrane glycoprotein, was selected as it is a well-known marker of tumor aggressiveness for both primary and metastatic CRC [32,33]. When evaluated in combination with CD133, ALDH1A1 has been reported to provide a more accurate assessment of tumor aggressiveness [34] and was, therefore, included in the panel of markers. Instead, given that intracellular SOX2 transcription factors and the associated surface marker VE-cadherin have been linked to increased metastatic potential in CRC [35,36,37], these markers were selected to investigate the potential of the treatments on markers of stemness and self-renewal. Relative CD133 (*PROM1*) expression at both mRNA and protein levels was first compared between untreated (CTRL) SW620 and Caco-2 primary spheroids after seven days (Figure 5A–C). Using SW620 as the calibrator (RQ = 1), CD133 mRNA levels were significantly lower in Caco-2 spheroids (Figure 5A). CD133 protein expression was assessed by Western blot analysis (Figure 5B), normalized to β-actin, and quantified using SW620 as the reference sample (set to 1); under these conditions, CD133 protein levels were comparable between the two cell types (Figure 5C). The effects of SPD (S), EUG (E) and SPD+EUG (S+E) on CD133 were then evaluated in comparison to the untreated CTRL (C) (set to 1) in each cell line (Figure 5D–I). In SW620 spheroids, CD133 mRNA level was not significantly altered by SPD or EUG alone, whereas SPD+EUG markedly reduced CD133 transcript levels (Figure 5D). In contrast, at the protein level, CD133 expression did not mirror the transcript pattern, as SPD+EUG-treated spheroids displayed elevated CD133 protein levels relative to the untreated CTRL (Figure 5E,F). In Caco-2 spheroids, CD133 mRNA levels were significantly reduced by all treatments (Figure 5G), and CD133 protein expression was also significantly decreased in comparison to the CTRL, with SPD+EUG producing the greatest reduction (Figure 5H,I).

**Figure 6:**
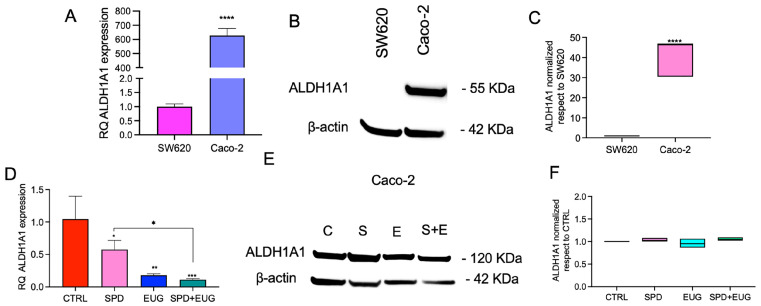
ALDH1A1 mRNA and protein expression in CRC spheroids. Panels (**A**–**C**) show the baseline comparison of transcript (RT-qPCR) and protein (Western blot) levels between untreated metastatic SW620 and primary Caco-2 first-generation spheroids after 168 h (SW620 set as calibrator = 1). Panels (**D**–**F**) illustrate the impact of 168 h exposure to SPD, EUG, and SPD+EUG on ALDH1A1 mRNA and protein expression specifically in the Caco-2 model. All results are relative to the untreated CTRL (calibrator = 1). Significant differences: * *p* < 0.05, ** *p* < 0.01, *** *p* < 0.001, **** *p* < 0.0001.

**Figure 7:**
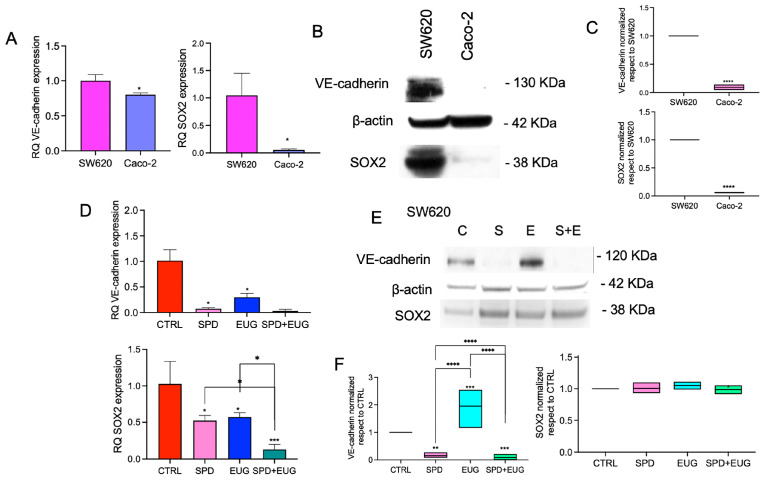
SOX2 and VE-cadherin mRNA and protein expression in CRC spheroids. Panels (**A**–**C**) show the baseline comparison of transcript (RT-qPCR) and protein (Western blot) levels between untreated metastatic SW620 and primary Caco-2 first-generation spheroids after 168 h (SW620 set as calibrator = 1). Panels (**D**–**F**) illustrate the impact of 168 h exposure to SPD, EUG, and SPD+EUG on SOX2 and VE-cadherin mRNA and protein expression specifically in the SW620 model, as these markers were not significantly expressed in Caco-2 cells. All results are relative to the untreated CTRL (calibrator = 1). Significant differences: * *p* < 0.05, ** *p* < 0.01, *** *p* < 0.001, **** *p* < 0.0001.

The efficacy of the treatments was then evaluated on CSC markers according to their expression in each cell line. Figure 6 shows the effects of SPD, EUG and SPD+EUG on ALDH1A1 expression in Caco-2 spheroids, whereas Figure 7 reports the effects of the treatments on SOX2 and VE-cadherin expression in SW620 spheroids. As in the previous analyses, treatment effects were quantified relative to the untreated CTRL, which was used as the calibrator and set to a value of 1 for both RNA (RQ) and relative protein expression. ALDH1A1 expression was first compared between untreated SW620 and Caco-2 spheroids after 168 h. RT-qPCR analysis showed ALDH1A1 mRNA expression (RQ), with Caco-2 displaying higher transcript levels than SW620 (Figure 6A). Figure 6B shows a representative Western blot for ALDH1A1 and β-actin in SW620 and Caco-2 cells. The subsequent densitometric quantification of ALDH1A1, normalized to β-actin and reported relative to SW620 as the reference, is presented in Figure 6C; these data confirm significantly higher ALDH1A1 protein level in Caco-2 cells compared to SW620. Due to the negligible baseline expression of ALDH1A1 in SW620 cells, the subsequent evaluation of SPD, EUG, and SPD+EUG effects on this marker was conducted exclusively in Caco-2 spheroids (Figure 6D–F). The effects of SPD, EUG and SPD+EUG on ALDH1A1 expression were then assessed in Caco-2 spheroids in comparison to the untreated CTRL. RT-qPCR showed that ALDH1A1 mRNA was reduced by the treatments, with the strongest reduction observed for the combined treatment (Figure 6D). Figure 6E shows a representative Western blot for ALDH1A1 and β-actin in Caco-2 spheroids under CTRL (C), SPD (S), EUG (E), and SPD+EUG (S+E) conditions. Densitometric quantification of ALDH1A1 normalized to β-actin (relative to CTRL) shows no significant change at the protein level across treatments (Figure 6F).

SOX2 and VE-cadherin expression was first compared between untreated SW620 and Caco-2 spheroids after 168 h. RT-qPCR analysis revealed significantly higher SOX2 mRNA (RQ) in SW620 spheroids compared with Caco-2 (Figure 7A). Similarly, VE-cadherin (*CDH5*) mRNA levels were significantly higher in SW620 than in Caco-2 (Figure 7B). Figure 7C shows representative Western blot images for VE-cadherin, SOX2, and β-actin in SW620 and Caco-2 cells, along with the corresponding densitometric analysis normalized to β-actin and reported relative to SW620. This analysis revealed significantly lower VE-cadherin and SOX2 protein levels in Caco-2 cells compared with SW620. Since SOX2 and VE-cadherin were not significantly expressed in Caco-2 cells, the analysis of treatment effects on these protein markers was focused solely on the SW620 model (Figure 7D–F). The effects of SPD, EUG and SPD+EUG were then evaluated in SW620 spheroids relative to untreated CTRL. RT-qPCR analysis revealed that VE-cadherin and SOX2 mRNA levels decrease following treatment, with the combined SPD+EUG condition showing the lowest SOX2 transcript levels (Figure 7D). Figure 7E shows a representative Western blot for VE-cadherin, SOX2, and β-actin in SW620 cells under CTRL (C), SPD (S), EUG (E), and SPD+EUG (S+E) conditions. Densitometric quantification (normalized to β-actin and relative to CTRL) shows an increase in VE-cadherin protein level in the EUG condition, whereas SOX2 protein levels do not show a marked decrease across treatments (relative to CTRL) in the quantification panel (Figure 7F).

### 2.4. Principal Component Analysis (PCA) of Cellular Responses to SPD, EUG and SPD+EUG

**Figure 8:**
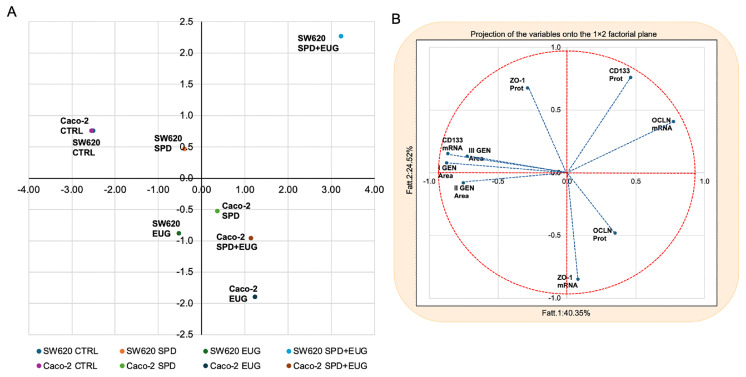
Principal Component Analysis (PCA) of CRC spheroids treated with SPD and EUG. (**A**) PCA score plot showing the distribution of samples along the first two principal components (PC1 and PC2), generated from a multivariate dataset including cancer stem cell (CSC)- and tight junction (TJ)-associated markers at both transcript and protein levels. The analysis includes metastatic SW620 and primary Caco-2 spheroids under CTRL, SPD, EUG, and SPD+EUG conditions. (**B**) PCA loading plot showing the projection of the variables included in the analysis onto the PC1–PC2 plane. The red dashed line represents the correlation circle, while the blue dashed arrows represent the variables included in the multivariate analysis. The variables considered were spheroid area (first to third generations, I–III GEN Area), mRNA and protein levels for CD133, ZO-1, and OCLN.

To obtain an integrated and exploratory overview of treatment-induced molecular responses, a Principal Component Analysis (PCA) was performed on datasets complete for all experimental conditions (CTRL, SPD, EUG and SPD+EUG), including both SW620 and Caco-2 spheroids. PCA is an unsupervised multivariate approach that reduces data dimensionality by identifying principal components explaining the largest proportion of total variance, without imposing predefined group classifications. The score plot reported in Figure 8A shows the distribution of samples along the first two principal components (PC1 and PC2). A clear separation between SW620 and Caco-2 spheroids is evident, indicating that cell line-specific molecular features represent a major source of variability within the dataset. Within each cell line, samples exposed to SPD, EUG and SPD+EUG consistently occupy regions of the PCA space that are distinct from the corresponding CTRL samples, indicating that all treatments induce a marked shift in the overall molecular profile relative to untreated conditions. Importantly, the untreated CTRL samples of the two cell lines cluster in the same PCA quadrant, indicating a similar baseline multivariate profile. The variables most strongly associated with this clustering are spheroid area across generations (I GEN Area, II GEN Area and III GEN Area), CD133 mRNA and, to a lesser extent, ZO-1 protein levels. This suggests that, for these parameters, the two cell lines display comparable baseline behavior despite their different biological origin. Notably, in Caco-2 spheroids, treatment-associated shifts appear more uniform, with SPD, EUG and SPD+EUG samples clustering relatively closely to one another and occupying a quadrant distinct from the CTRL samples. This indicates a consistent treatment-associated response in this cell line. The variables most strongly associated with the treated Caco-2 samples are OCLN protein and ZO-1 mRNA expression, suggesting that tight junction-related markers represent key contributors to the treatment-associated molecular shift in these spheroids. In contrast, SW620 spheroids display a more heterogeneous response, with SPD-, EUG- and SPD+EUG-treated samples occupying more clearly separated regions of the PCA space, indicating differential molecular effects induced by the individual treatments and their combination. In particular, SPD and EUG treatments are associated with a reduction in spheroid recurrence compared to the untreated CTRL and display a response pattern that is broadly similar to that observed in Caco-2 spheroids. The SPD+EUG condition shows a distinct positioning compared with single treatments, indicating a treatment-specific response of metastatic spheroids. In this condition, the variables most strongly contributing to sample positioning are OCLN mRNA and CD133 protein expression. Considering that CD133 is a marker associated with cancer stemness, these findings suggest that, in SW620 spheroids, treatments may reduce recurrence while modulating stemness-related molecular features. The corresponding loading plot shown in Figure 8B illustrates the projection of individual molecular markers onto the principal component space and highlights their differential contribution to sample distribution. The markers are positioned in distinct regions of the plot, indicating heterogeneous contributions to the principal components. Transcript- and protein-level measurements of the same markers do not always colocalize, reflecting a partial dissociation between mRNA expression and protein abundance. This divergence is consistent with the univariate analyses and suggests the involvement of post-transcriptional or post-translational regulatory mechanisms. Moreover, differences in variable positioning between SW620 and Caco-2 datasets underscore cell line-specific molecular architectures and differential responsiveness to the treatments. Overall, the PCA reveals structured patterns in the dataset, highlighting both cell line-dependent baseline differences and treatment-associated shifts in the multivariate molecular profiles.

## 3. Discussion

The adoption of plant-derived compounds with anti-tumorigenic efficacy is gaining interest due to the adverse effects of conventional therapies. Within this framework, the safety and selective toxicity of SPD and EUG have been previously established using the non-malignant NCM460 human colon mucosal epithelial cell line [22,38]. Prior evidence demonstrated that EUG triggers apoptosis selectively in malignant lines while sparing healthy cells. Furthermore, in 3D co-culture models, the SPD+EUG combination was shown to actively support the structural integrity of healthy enterocytes by enhancing OCLN expression and promoting protective autophagic flux [22,38]. Complementing this preservation of healthy tissue, SPD+EUG simultaneously exerts a targeted anti-tumorigenic action; specifically, it was previously shown to induce a synergistic effect on increasing apoptotic activity (caspase-3) and decreasing migration percentage in the SW620 cell line [22]. The hypothesis was that the efficacy of SPD+EUG (administered to first-generation spheroids at the time of seeding) would also be reflected by increased TJ and decreased CSC gene expression. To verify this hypothesis, the preventive anti-CRC potential of exogenous SPD+EUG, as well as the interplay between SPD and EUG, on CSC marker and TJ protein expression in relation to spheroid formation capacity were investigated. To the best of our knowledge, the anti-CRC potential of SPD on TJ and CSC marker expression has not been investigated previously. Thus, the present study aimed to contribute to the growing body of knowledge on the role of SPD in cancer [27], as well as on the preventive potential of natural products in targeting CSCs and the tumor microenvironment (TME). The efficacy of SPD and EUG (alone and in combination) in reducing spheroid area was first validated after 168 h in first-generation SW620 and Caco-2 spheroids. The present results corroborated the size reduction reported within the 96 h time frame analyzed in our previous work, which was attributable to increased apoptosis [22]. Treatment efficacy in terms of self-renewal capacity, involving the reduction in both spheroid number and size over subsequent generations, was further supported by the significant reduction in second-generation SW620 and Caco-2 spheroid area (to approximately 5–10% of the respective CTRLs), as well as by the marked decrease in the number of third-generation SW620 spheroids. Notably, only 17% of the original primary SW620 spheroids treated with SPD+EUG retained the capacity for tertiary spheroid formation. Of particular interest was the significantly smaller spheroid area observed following SPD+EUG administration compared to SPD and EUG alone in first-generation spheroids, together with the pronounced additional reduction in spheroid area observed in all treated second-generation spheroids. These findings prompted the question of whether, and to what extent, TJ and CSC expression was altered in primary spheroids following treatment exposure. Prior to addressing treatment effects, baseline TJ and CSC expression profiles were therefore examined in untreated SW620 and Caco-2 spheroids.

From the present results, OCLN and ZO-1 RNA transcript expression, together with OCLN and ZO-1 protein levels, were significantly higher in Caco-2 than in SW620 spheroids. The overall lower TJ gene expression observed in SW620 spheroids corroborates previous reports documenting reduced expression of OCLN [16,18] and CLDN1 [15,39,40,41] in aggressive, less differentiated metastatic CRC cell types. These findings support the use of the SW620 and Caco-2 spheroid models to investigate differential TJ regulation in relation to tumor aggressiveness. Beyond their canonical role in maintaining epithelial barrier integrity, tight junction proteins such as OCLN and ZO-1 are increasingly recognized as active regulators of intracellular signaling pathways involved in epithelial differentiation, cellular plasticity and tumor progression [42]. In particular, ZO-1 functions as a multifunctional scaffold protein linking transmembrane TJ components to the actin cytoskeleton and to transcriptional regulators, thereby influencing gene expression programs associated with epithelial identity and proliferation [18,43]. Alterations in ZO-1 expression and subcellular localization have been reported to accompany EMT and to promote invasive and stem-like phenotypes in colorectal and gastrointestinal cancers [17,44]. Similarly, loss or downregulation of OCLN has been consistently associated with reduced epithelial differentiation, increased tumor aggressiveness and unfavorable prognosis across multiple tumor types, including colorectal cancer [16,18]. Although treatment-induced modulation of OCLN and ZO-1 transcripts was limited, protein-level changes were evident and appeared to be cell type- and treatment-dependent. This dissociation between mRNA and protein expression suggests that TJ remodeling in response to SPD and EUG may be predominantly regulated at the post-transcriptional or post-translational level, potentially involving protein stability, trafficking or junctional assembly rather than transcriptional control [18]. Importantly, modulation of TJ protein expression may contribute to the observed reduction in spheroid growth and self-renewal capacity by reinforcing epithelial features and counteracting EMT-associated stemness programs. Taken together, these findings support the concept that modulation of tight junction components and CSC-associated markers represents interconnected rather than independent processes, with TJ remodeling contributing to the regulation of epithelial plasticity and stem-like properties in colorectal cancer spheroids [17,18]. Overall, the PCA highlights structured patterns in the dataset, revealing both cell line-dependent baseline differences and treatment-associated molecular changes. By integrating CSC- and TJ-related markers at both the transcript and protein levels, this multivariate analysis supports the conclusion that SPD and EUG, either alone or in combination, modulate colorectal cancer spheroids through complex and cell type-dependent molecular responses. Notably, the PCA suggests that treatment-induced reductions in spheroid recurrence may be accompanied by differential modulation of stemness-related markers in metastatic SW620 spheroids, underscoring the cell line-specific nature of the observed responses. With respect to CSC markers, CD133 expression was detected in both SW620 and Caco-2 spheroids, whereas ALDH1A1 expression was predominantly associated with Caco-2 spheroids. ALDH1A1 mRNA and protein levels were markedly higher in untreated Caco-2 spheroids compared to SW620, consistent with the association of ALDH1A1 with a more differentiated epithelial phenotype. Following treatment, ALDH1A1 transcript levels in Caco-2 spheroids were significantly reduced by SPD, EUG and SPD+EUG, with the combined treatment inducing the strongest reduction. However, despite this consistent decrease at the mRNA level, ALDH1A1 protein expression was not significantly altered by any of the treatments. This dissociation between transcript and protein expression suggests post-transcriptional regulation and indicates that short-term modulation of ALDH1A1 transcription does not necessarily translate into immediate changes in protein abundance [45]. SOX2 and VE-cadherin expression was predominantly associated with SW620 spheroids, in agreement with their reported roles in promoting stemness, self-renewal and metastatic potential in CRC [35,36,37]. In SW620 spheroids, SOX2 mRNA expression was significantly reduced by all treatments, with SPD+EUG inducing the most pronounced decrease. However, SOX2 protein levels did not show a consistent reduction relative to the CTRL across treatments, indicating that transcriptional downregulation was not fully reflected at the protein level within the experimental time frame. Similarly, VE-cadherin mRNA expression was significantly reduced following treatment, whereas VE-cadherin protein expression did not decrease correspondingly and was increased following EUG treatment. Together, these findings highlight a divergence between transcript and protein regulation for CSC-associated markers, suggesting that post-transcriptional mechanisms modulate protein expression in treated spheroids and reflect the complex regulatory landscape of CRC cells. In mammalian systems, mRNA abundance typically explains only about 40% of the variation in protein levels, with the remainder governed by translation efficiency and protein degradation rates [45]. Specifically, for TJs like OCLN and ZO-1, a similar divergence has been documented in colorectal metastases, where high protein stability and low turnover within junctional complexes allow proteins to remain abundant despite decreased transcript levels [44]. Regarding CSC markers, SPD may further influence this balance through the hypusination of eukaryotic translation initiation factor 5A (eIF5A), which requires this unique spermidine-dependent modification to facilitate the synthesis of proteins vital for mitochondrial function, autophagy, and cellular survival [27]. This SPD-dependent mechanism fine-tunes protein synthesis, potentially prioritizing the translation of key survival and stemness factors even when their corresponding transcripts are downregulated. Previous studies have reported that EUG can reduce CSC marker expression and sphere-forming capacity in various cancer models. In breast cancer (MCF-7), EUG was shown to reduce tertiary sphere formation and CSC marker expression [46]. In CRC, EUG at 500 µM reduced CD133 expression in HCT-116 cells [29], while also modulating the expression of tumor suppressor and oncogenic genes [47]. More recently, a platinum (IV) complex incorporating EUG was reported to induce a synergistic downregulation of ALDH and CD133 in SW680 spheroids, accompanied by a reduction in spheroid area [30]. In the present study, the significant reduction in first-generation spheroid area following SPD+EUG administration was associated primarily with decreased CD133 protein expression and with reduced transcript levels of SOX2 and VE-cadherin, whereas corresponding protein-level changes were not consistently observed for all CSC markers.

These findings may reflect a temporal divergence in the molecular response to SPD+EUG, characterized by an initial downregulation of transcript levels followed by a more gradual depletion of the corresponding protein pool. In this context, the observed dissociation could be interpreted through the framework of SPD acting as a translational regulator or “rheostat” [45]. By modulating protein synthesis efficiency through eIF5A hypusination, SPD might temporarily prioritize the translation of key survival factors, potentially explaining why certain CSC markers remain detectable in primary spheroids despite significant transcriptional suppression [27]. This observation is consistent with dynamics reported in other CRC models, where proteins within stable junctional complexes or specific subpopulations remain abundant despite a drop in their transcripts [44]. Ultimately, this translational fine-tuning appears insufficient to sustain the CSC phenotype in the long term, as evidenced by the subsequent collapse of self-renewal capacity observed in tertiary generations [22], likely following the eventual exhaustion of essential stemness factors such as CD133 and SOX2 [32,37]. While this interpretative framework offers a plausible explanation for the observed dynamics, the biochemical mechanisms underlying the individual and combined effects of SPD and EUG were not investigated in the present study and warrant further examination. In particular, it will be important to assess whether the transcript-level changes observed in primary spheroids are reflected at the protein level in second- and third-generation spheroids. For homotypic spheroids, exogenous SPD administration, both alone and in combination with EUG, was shown to exert anti-CRC effects on CSC marker gene expression. Given the reported pro-tumorigenic role of endogenous SPD, in contrast to the potential anti-tumorigenic effects of exogenous SPD administration [27], polyamine metabolism will be an important area of future research. Moreover, recent findings in glioblastoma have demonstrated that exogenous SPD administration in an endogenous SPD-producing TME can promote tumor aggressiveness in an immune-dependent manner via reduced CD8+ T-cell activity [48]. These observations underscore the importance of incorporating immune components into future preclinical models to fully evaluate the effects of SPD on CRC progression.

To properly contextualize the translational value of these results, it is essential to explore the potential practical applications in a clinical setting. Given its ability of the treatments to modulate stemness markers and reduce spheroid recurrence, our evidence suggests that EUG could be effectively utilized as an adjuvant alongside conventional cytostatic drugs [23,24]. Since resistant CSC subpopulations are primarily responsible for treatment failure and relapse in CRC [32,34,36], the finding that these bioactive compounds can impair their self-renewal capacity provides a compelling and highly relevant future direction for this research. Specifically, it would be of interest to investigate whether this combination could sensitize resistant cancer stem cell subpopulations to standard chemotherapy [23,30], potentially improving therapeutic efficacy while maintaining the safety profile for healthy tissues already observed in our models [22,38].

## 4. Materials and Methods

### 4.1. Cell Lines, Bioactive Compound Sources and Culture Conditions

Two human CRC cell lines, Caco-2 and SW620, were used. The primary colorectal adenocarcinoma, epithelial Caco-2 cell line (AddexBio C0009009; San Diego, CA, USA) was cultured with Eagle’s Minimum Essential Medium (EMEM; AddexBio, San Diego, CA, USA), supplemented with 20% Fetal Bovine Serum (FBS; GIBCO, Waltham, MA, USA) and 1% penicillin–streptomycin (Pen/Strep; GIBCO). The lymph node metastasis cell line, SW620, purchased from the American Type Culture Collection (AddexBio C0009002; San Diego, CA, USA), was cultivated in high-glucose Dulbecco’s Modified Eagle Medium (DMEM; GIBCO) containing 10% Fetal Bovine Serum (FBS; GIBCO), 1% penicillin–streptomycin (Pen/Strep; GIBCO) and 1% L-glutamine (GIBCO). Details pertaining to the culture conditions were provided previously [49].

SPD was obtained from wheat germ, supplied by Molino Naldoni milling company (Faenza, Italy), as indicated in D’Amen et al. [50], and EUG was provided from clove oil (Eugenia spp.; Sigma-Aldrich, St. Louis, MO, USA). The polyamine content of the wheat germ was determined by HPLC-ESI-MS using a Waters e2695 separation module equipped with an Acquity QDa detector (Waters Corp., Milford, MA, USA) and was reported to contain SPD (750 mg/kg), putrescine (170 mg/kg), and spermine (325 mg/kg) [38]. Given that SPD was the major constituent, the wheat germ component was referred to as SPD. Dose-dependent responses of SPD and EUG on the proliferation of Caco-2 and SW620 spheroids was determined in the previous work [22]. The standard concentrations administered in the present study were the same as those of the previous study [22] and were as follows: 150 µM SPD, 100 µM EUG and 150 µM SPD + 100 µM EUG.

### 4.2. Generation of 3D Spheroids Using SW620 and Caco-2 Cell Lines and Treatment with SPD and EUG Alone and in Combination

Homotypic 3D spheroid cultures were obtained using the liquid overlay method [51]. Tissue culture (96-well) plates were coated with 100 μL 1.5% agar (UltraPure™ Agarose; Invitrogen, Thermo Fisher Scientific, Waltham, MA, USA) dissolved in DMEM, and the polymerized agar irradiated with UVB for 20 min. Thereafter, SW620 (1000 cells/well) and Caco-2 cells (1000 cells/well) were respectively seeded, and spheroid formation was allowed to proceed for 168 h at 37 °C. An initial study was performed to assess the effects of SPD+EUG administration on spheroid area and proliferation either after or before spheroid formation. To this end, SPD+EUG was administered either 24 h after seeding (at which point spheroid development was already evident) or at the time of seeding. Since it was established that SPD+EUG exercised a greater anti-CRC impact on spheroid area and proliferation when administered at the time of seeding, all subsequent experiments were performed by including both SPD and EUG individually and in combination at the time of seeding. CTRL contained only ethanol and DMEM. After 168 h, Caco-2 and SW620 primary spheroid cultures were harvested. The first-generation spheroids were used to examine the capacity to form subsequent sphere generations. TJ marker and CSC marker gene expression (RNA transcript and protein expression) analyses were performed after 168 h in the first-generation spheroids.

### 4.3. Secondary, Tertiary and Quaternary Sphere Formation Assays

First-generation spheroids were cultivated as recorded above. After seven days, the number of primary-generation spheroids was counted. Subsequently, the spheroids were dissociated enzymatically with 0.05% trypsin and 0.02% EDTA in PBS. The resulting single cells were replated on agar-pretreated 96-well plates containing DMEM medium at the same densities and culture conditions used for the first-generation spheroids and incubated to allow the formation of the next generation. The spheroids were counted after seven days and then subjected to further sequential dissociation and reseeding passages for a further two consecutive generations. The same procedure was adopted to monitor the self-renewal capacity of both SW620 and Caco-2 spheroids. After each generation, the spheroid area was assessed, as described in Section 4.4. 

### 4.4. Determination of Spheroid Area and Viability (MTT) Measurements

The anti-proliferative effect of SPD, EUG and SPD+EUG on SW620 and Caco-2 spheroids was evaluated by calculating the total spheroid area as described previously [22]. Briefly, total spheroid areas were examined under an optical microscope (Eclipse Ts2, Nikon Corporation, Tokyo, Japan) at a magnification of ×4 or ×10 and photographed. The photographs were then examined using ImageJ software (Wayne Rasband, version 2.9.0/1.53t; National Institute of Mental Health, Bethesda, MD, USA), and the images processed to pixels (300 pixels/2.54 cm). The spheroid areas were calculated for all 168 h old treated and CTRL spheroids for each consecutive generation, respectively. Spheroid areas for each treatment were expressed as a percentage of the untreated CTRL.

Cell viability of the 3D spheroids was measured using the MTT assay (Life Technologies, Carlsbad, CA, USA), according to the method of Saltari et al. [52].

### 4.5. Reverse Transcription–Quantitative Polymerase Chain Reaction (RT-qPCR) of Tight Junction (TJ) and Cancer Stem Cell (CSC) Marker Genes 

RT-qPCR analysis was performed as a two-step reaction. Total RNA was extracted from Caco-2 and SW620 cells using RNA Extracol (EURx, Gdańsk, Poland), followed by the TransZol Up RNA Kit (TransGen Biotech, Beijing, China), and quantified using a NanoDrop spectrophotometer (Thermo Fisher Scientific, Waltham, MA, USA). cDNA was synthesized from 1 µg of total RNA using the Hiscript^®^ III RT SuperMix for qPCR (+gDNA wiper) (Vazyme, Nanjing, China) in a T-Gradient Thermoblock (Biometra GmbH, Göttingen, Germany). Quantitative PCR was conducted on a StepOne Real-Time PCR System (Applied Biosystems, Thermo Fisher Scientific, Waltham, MA, USA) using Power SYBR Green PCR Master Mix (Applied Biosystems, Waltham, MA, USA). The expression of target genes (*ALDH1A1*, *CDH5*, *PROMP1*, *SOX2*, *OCLN*, and *TJP1*) was normalized to β-Actin (*ACTB*) as the reference gene. Primer sequences are detailed in Appendix A. Relative fold changes in gene expression were calculated using the 2^−ΔΔCt^ method, with untreated cells or specific cell types used as calibrators.

### 4.6. Protein Extraction and Western Blot

SW620 and Caco-2 spheroids were collected, centrifuged at 1200 rpm for 5 min, and the resulting pellets were lysed in radioimmunoprecipitation assay buffer (RIPA) lysis buffer (Thermo Fischer Scientific). Protein concentration was measured using the Pierce™ BCA Protein Assay Kit (Thermo Fischer Scientific), according to the manufacturer’s instructions. Equal amounts of protein were loaded onto Invitrogen™ Bolt™ 4–12% Bis-Tris Plus WedgeWell™ gels (Thermo Fischer Scientific). Protein transfer onto membranes was performed using the iBlot 2 Dry Blotting System (Thermo Fischer Scientific), following the manufacturer’s instructions. Membranes were blocked with EveryBlot Blocking Buffer (Bio-Rad Laboratories, Hercules, CA, USA); primary antibodies for the selected CSC and TJ proteins of interest (Appendix B) were incubated in blocking buffer, followed by incubation with secondary antibodies conjugated with HRP (Appendix B). Chemiluminescence detection was performed using SuperSignal™ West Pico PLUS Chemiluminescent Substrate (Thermo Fisher Scientific, Waltham, MA, USA). Protein bands were acquired through digital imaging or conventional film development. Digital signals were captured using the ChemiDoc MP Imaging System (Bio-Rad Laboratories, Inc., Hercules, CA, USA), while manual acquisition was conducted in a darkroom by developing signals on CL-XPosure™ Films (5 × 7 inches; Thermo Fisher Scientific). Films were processed using EMS Replacement for Kodak Developer D-19 (Electron Microscopy Sciences, Hatfield, PA, USA) and Kodak Professional Fixer. Regardless of the acquisition method, band intensities were quantified through densitometric analysis using ImageJ software (Wayne Rasband, version 2.9.0/1.53t; National Institute of Mental Health, Bethesda, MD, USA), with all protein levels normalized to β-actin as the internal reference control.

### 4.7. Statistical Analyses

All experiments were performed in triplicate. The data were expressed as mean values of three different experiments. Prior to applying parametric tests, the assumptions of data normality and homogeneity of variance were confirmed. Statistical analysis was conducted using GraphPad Prism Version 10.2.3 (2024). One-way variance (ANOVA) was used to determine any significant differences between the respective treatments and the CTRL. Using Dunnett’s multiple comparisons test, significant differences were represented as follows: ns * *p* < 0.05, ** *p* < 0.01, *** *p* < 0.001, and **** *p* < 0.0001. In the graphs, only mean values expressed with stars were statistically different. Using one-way ANOVA, significant differences between SPD (150 µM) and EUG (100 µM) alone and SPD+EUG (150 µM SPD + EUG 100 µM) for each of the variables studied were analyzed by one-way ANOVA and the Turkey–Kramer test at the 95% confidence level (*p* < 0.05).

Principal Component Analysis (PCA) was performed as an unsupervised multivariate approach to explore relationships among samples and variables and to reduce data dimensionality. The analysis was conducted on datasets complete for all experimental conditions (CTRL, SPD, EUG and SPD+EUG) and included both SW620 and Caco-2 spheroids. The variables included in the PCA were spheroid area of the first (I GEN), second (II GEN) and third (III GEN) generations; CD133 protein expression and mRNA levels; ZO-1 protein expression and mRNA levels; and OCLN protein expression and mRNA levels. Prior to analysis, data were standardized to ensure equal weighting of variables. PCA was performed using Statistica software (version 7.1; StatSoft, Tulsa, OK, USA). The results were visualized using score plots, representing the distribution of samples, and loading plots, representing the contribution of individual variables, based on the first two principal components.

## 5. Conclusions

In this study, SPD and EUG, particularly in combination, significantly reduced spheroid growth and self-renewal capacity in primary and metastatic colorectal cancer spheroid models. Distinct baseline expression patterns of tight junction and cancer stem cell-associated markers reflected differences in tumor origin and differentiation status. Treatment-induced modulation of CSC- and TJ-related transcripts was observed but was not consistently mirrored at the protein level, highlighting the relevance of post-transcriptional regulatory mechanisms. These findings support the use of three-dimensional spheroid models for integrated molecular profiling and underscore the importance of combining transcriptomic and proteomic analyses when evaluating bioactive compounds with potential relevance for colorectal cancer treatment and prevention.

## Data Availability

The original contributions presented in this study are included in the article. Further inquiries can be directed to the corresponding author.

## Abbreviations

The following abbreviations are used in this manuscript:

ALDH1A1	Aldehyde Dehydrogenase 1 Family Member A1
CCSC	Colorectal CSC
CD133	Prominin-1
CSC	Cancer Stem Cells
CRC	Colorectal Cancer
CTRL	Control
EMT	Epithelial–Mesenchymal Transition
EUG	Eugenol
OCLN	Occludin
SOX2	SRY-box transcription factor 2
SPD	Spermidine
TJ	Tight Junctions
TME	Tumor Microenvironment
VE-cadherin	Vascular Endothelial-Cadherin
ZO-1	Zonula Occludens-1
I GEN	First-Generation
II GEN	Second-Generation
III GEN	Third-Generation
IV GEN	Fourth-Generation

## Appendix A

**Table A1:** Primers used for reverse transcription–quantitative polymerase chain reaction (RT-qPCR).

Gene Groups	Gene Name	Primer Sequences (5′ → 3′)
Stemness genes	*ALDH1A1*	F-AGCCTTCACAGGATCAACAGA R-GTCGGCATCAGCTAACACAA
	*PROM1* (CD133)	F-CTGGGGCTGCTGTTTATTATTCTG R-ACGCCTTGTCCTTGGTAGTGTTG
	*SOX2*	F-GCCGAGTGGAAACTTTTGTCG R-GCAGCGTGTACTTATCCTTCTT
Epithelial–mesenchymal transition (EMT) genes	*CDH5* (VE-cadherin)	F-CCTGACTGTGGAGGCCAAAGA R-TTCTCACACACTTTGGGCTGGTAG
Functional genes	*OCLN*	F-AATTGGTCACCGAGGGAGGA R-ATAAACCAATCTGCTGCGTCC
	*TJP1* (ZO-1)	F-AGCCATTCCCGAAGGAGTTG R-AGGATCACAGTGTGGTAAGCG
Housekeeping genes	*ACTB* (β-Actin)	F-GAAGGATTCCTATGTGGGCG R-TCATTGTAGAAGGTGTGGT

## Appendix B

**Table A2:** Antibodies used for Western blotting.

Antibody	Isotype	Host	Dilution	Company
ALDH1A1	IgG	Rabbit	1:1000	ABclonal
CD133	IgG	Rabbit	1:1000	ABclonal
SOX2	IgG	Rabbit	1:2000	ABclonal
VE-cadherin	IgG	Rabbit	1:500	ABclonal
OCLN	IgG	Mouse	1:1000	Novus Biologicals
ZO-1	IgG	Rabbit	1:1000	Invitrogen
HRP	IgG	Goat-antiMS	1:10,000	Thermo Fischer
HRP	IgG	Goat-antiRB	1:10,000	Novex
β-Actin	IgG	Rabbit	1:2000	Novus Biologicals
β-Actin	IgG	Mouse	1:400	Novus Biologicals

HRP = horse radish peroxidase. The dilutions of the HRP Goat antiMS and HRP Goat antiRB for β-Actin were 1:5000 and 1:20,000, respectively.
